# Rational resistance amidst gentle breeze and mild rain: Examining online collective behavior among the Chinese public using the elaborated social identity model

**DOI:** 10.1371/journal.pone.0303603

**Published:** 2024-05-24

**Authors:** Wang Qing, Zhang Xuebo

**Affiliations:** School of Information Technology in Education, South China Normal University, Guangzhou, China; Tecnologico de Monterrey, MEXICO

## Abstract

This research examines internet collective behavior in mainland China during the COVID-19 pandemic, focusing on the factors and characteristics that drive such behavior. The Chinese government initially implemented a conservative and biased policy to contain the spread of the virus, but the sudden lifting of lockdown measures in late 2022 resulted in a surge in infections and scarcity of medical resources. This policy shift led many Chinese internet users to perceive the government’s actions as hasty and harsh, prompting them to engage in collective online behavior. The study employed a survey-based approach, collecting 1,626 valid questionnaires, which underwent reliability testing, descriptive statistical analysis, and a difference-in-differences test. A structural equation model (SEM) was then constructed and applied to comprehensively analyze the mediating and moderating effects of latent variables. Ethical considerations were prioritized, with informed consent obtained from all participants, who were provided with detailed information about the study and given sufficient time to review and ask questions. The research yielded three primary conclusions: the Chinese public demonstrated a perception of fairness and exhibited obedience, respect, and cooperation with the government during the epidemic; the observed online collective behavior can be characterized as a moderate and rational form of resistance, explained by the elaborated social identity model (ESIM); and the middle class consistently adopted a self-vulnerability strategy, positioning themselves as beneficiaries of protection to maximize their own interests in epidemic prevention and control. This study shows notable insights into internet collective behavior in mainland China during the COVID-19 pandemic, highlighting perceptions, resistance, and strategies adopted by different segments of the population.

## 1. Introduction

The advent and widespread adoption of modern technology have ushered in a multitude of advantages and disadvantages. Within the realm of cyberspace, the complexities of social realities have given rise to frequent occurrences of online collective behavior among the Chinese public. This phenomenon can be attributed to various practical factors [1, 2]. Firstly, the emergence of the network society has fundamentally transformed power dynamics, both among individuals and those occupying positions of authority. The traditional dynamics pertaining to production, experience, power, and culture in human societies have undergone significant alterations due to the prevalent logic of networking [3–5]. A pivotal year in which internet-based power brought about changes in China’s physical realm was 2003. Influential internet clusters within the country, exemplified by incidents such as the Sun Zhigang incident, the SARS incident, and the BMW case, compelled the Chinese government to implement reforms aimed at rectifying unjust rules and regulations [6, 7]. Secondly, mainland China is presently undergoing a social transition characterized by a fusion of traditional and modern elements. Group events have become increasingly common, exhibiting regular fluctuations in frequency. These events are typically driven by interest-based demands, employ escalating strategies, yield amplified impacts, and frequently give rise to conflicts, particularly in the southern region [7–9].

Xue et al. [10] shows the social psychological principles governing Chinese individuals’ participation in collective action, offering insights into the motivations and factors driving their engagement in collective behavior. Building upon the Elaborated Social Identity Model (ESIM), our research extends this inquiry by examining the specific characteristics and factors influencing online collective behavior in mainland China in response to the swift easing of epidemic restrictions [10].

Many researchers introduced [11–14] that as societal transformation deepens, a growing division among social classes and a "rupture" in the social structure occur. While individuals in the upper strata of society are more likely to exert influence on public policy, those in the lower strata experience fragmentation and rely on collective power to assert their interests due to their limited individual influence. As a result, a series of group conflicts ensue [15–17]. In recent years, there has been increasing scholarly interest in understanding the dynamics of online collective behavior, particularly within the Chinese context [18, 19]. This literature review aims to provide a comprehensive analysis of previous studies and identify the gaps and limitations that our research seeks to address. Expanding upon this perspective, our study investigates the rational resistance displayed by the Chinese public in the online domain following the relaxation of COVID-19 lockdown measures [20–24].

Building upon this reflection, our research utilizes the ESIM to analyze the moderate and rational resistance exhibited by the Chinese public following the sudden lifting of COVID-19 lockdown measures. This study identifies research gaps that require attention. Firstly, the limited data sources used for constructing the Structural Equation Model (SEM), with only 30 references from the literature review, necessitates the inclusion of more diverse and validated data to enhance model robustness. Secondly, the study’s optimization analysis was constrained by a limited number of variables, emphasizing the need to incorporate additional relevant variables for a more comprehensive analysis [25–27]. In the preceding three years, the Chinese government has adopted a cautious approach to curb the spread of epidemics, which has had repercussions on the local economy. Nevertheless, in November 2022, the government introduced measures to ease prevention policies, subsequently leading to a significant surge in COVID-19 cases and placing strain on healthcare resources. The swift relaxation of restrictions drew criticism from Chinese internet users, resulting in a surge of negative sentiment and an increase in online collective behavior. This research study aims to examine the public’s involvement in online collective behavior as a response to changes in epidemic control measures in China. By taking into account social stability and comprehending the psychology of online communities, the study seeks to analyze the mechanisms that influence such behavior.

This article investigates the intentions and demands of the Chinese public in response to the sudden relaxation of epidemic control measures in late 2022. It addresses three research questions: Q1) What are the genuine intentions and demands of the Chinese public in response to the relaxation of measures? Q2) What factors influence the online collective behavior of the Chinese public in this incident, and what is the specific relationship between these factors? Q3) Can the ESIM explain the online collective behavior exhibited by the Chinese public in this context? By analyzing surveys and online expressions, this study aims to uncover the motivations and factors driving the collective behavior of the Chinese public during policy shifts in the COVID-19 pandemic. The ESIM can be used as a theoretical framework to understand the mechanisms shaping online collective behavior. The results can contribute to our understanding of the complex dynamics between public responses, social identity, and policy changes, offering insights for policymakers and researchers. The outcomes of this study possess the potential to yield valuable insights for guiding and fostering positive and secure online collective behavior in developing nations. The study focuses on the novel aspects and objectives involved in examining online collective behavior among the Chinese population in response to the sudden lifting of COVID-19 lockdown measures. Employing the ESIM, the study investigates the characteristics and factors that drive internet-based collective behavior in mainland China, with a particular emphasis on the middle class’s adoption of a self-vulnerability strategy in epidemic prevention and control. The objective of the research is to evaluate and analyze the influences on online collective behavior, aiming to understand the rational resistance observed and its specific manifestations within the Chinese context. The findings of this study have the potential to contribute to a comprehensive understanding of online collective behavior and provide valuable insights to guide and promote positive and secure online collective behavior in developing countries.

## 2. Literature review

### 2.1 Cross-cultural collective behavior analysis

During the 17th century, scholars and researchers from various disciplines in the Western context began showing interest in collective behavior. [28–32]. Building upon Durkheim’s ideas, Le Bon introduced the term "Crowd Behavior" to describe impulsive and irrational behavior resulting from emotional contagion among the masses [33, 34]. Popenoe suggested that group behavior arises due to shared influences or stimuli, resulting in scenarios characterized by unpredictability, disorganization, and instability [7].

In contemporary social psychology research, Wright et al. [8] proposed a widely accepted definition of collective behavior, stating that an individual can be considered engaged in collective behavior if they belong to a group and participate in actions aimed at improving the group’s status quo [8]. This definition emphasizes both the purpose of improving the group’s current situation and the interaction among individuals within the group. These definitions by Western scholars highlight common features of collective behavior, including disorganization, spontaneity, emotional contagion, interactivity between in-groups and out-groups, and the purposefulness of improving the group’s current situation. While research on collective behavior in mainland China has been relatively delayed, the usage of terms such as cluster behavior, group events, group emergencies, collective action, and group activities has been mixed and sometimes incorrect. These concepts have often been portrayed as a microcosm of people’s internal conflicts, with a general emphasis on political and rule of law aspects.

Wang et al. [11] posit that an excessive focus on the harmfulness and illegality of mass incidents tends to obscure the genuine needs of the people. However, this argument lacks empirical support and theoretical grounding, thus necessitating a critical examination in light of practical experiences and academic theories. Likewise, the study of networked group behavior and related concepts in mainland China demonstrates significant heterogeneity, which can be attributed to the conceptual ambiguity surrounding cluster behavior. Chinese scholars predominantly adhere to a research paradigm rooted in crisis management within the public domain, defining online group behavior as a social crisis associated with negative elements such as violence, crime, and disruption of social harmony. Nevertheless, this characterization fails to align with the observed characteristics in specific instances of research. In other words, only a few incidents exhibit features of group violence and law-breaking, while the majority of incidents involve the aggregation of public opinion and the expression of views. The act of online grouping is not strictly a social crisis. Consequently, the stance and premise of mainland China’s crisis management paradigm for online group behavior need to be reevaluated and reexamined. Scholars have made significant efforts in this regard, with some modifying the concept of network mass events while retaining its core [12–16]. Others have employed alternative concepts to study the phenomenon [17–21], while some argue that network mass events are essentially a subset of networked collective behavior and should be included in the discussion, potentially replacing networked collective behavior directly. Reflections on and revisions of the concept of online mass events have revealed a shift towards a neutral and scientific definition. Networked group events are inherently neutral, lacking a preconceived notion of good or bad, and require more objective and value-neutral concepts to encapsulate the phenomenon of netizen aggregation in virtual communities. This research posits that online collective behavior has its own core, involving the spontaneous, objective, and neutral collective efforts of the public, whether mild or intense, to draw the attention of authorities. During the 17th century, scholars from various disciplines in the Western context began showing interest in collective behavior. Building on Durkheim’s ideas, Le Bon introduced the term "Crowd Behavior" to describe impulsive and irrational behavior resulting from emotional contagion among the masses [6].

Popenoe suggested that group behavior arises from shared influences or stimuli, resulting in scenarios characterized by unpredictability, disorganization, and instability [7]. In contemporary social psychology research, Wright et al. [8] proposed a widely accepted definition of collective behavior, stating that an individual can be considered engaged in collective behavior if they belong to a group and participate in actions aimed at improving the group’s status quo [8]. This definition emphasizes the purpose of improving the group’s situation and the interaction among group members. These Western scholars’ definitions highlight common features of collective behavior, including disorganization, spontaneity, emotional contagion, interactivity between in-groups and out-groups, and the purpose of improving the group’s situation [35–38].

### 2.2 Cross-cultural behavior determinants

This study examines the factors contributing to the emergence of collective behavior, encompassing five major orientations: psychological, rational, structural, cultural, and integrative. Within the psychological orientation, concepts such as the "Circular Reaction theory," "Imitation theory," "Relative Deprivation theory," and "Value-added theory" are explored, drawing from theoretical studies rooted in social psychology, including Gustave Le Bon’s "the law of mental unity." The rational orientation investigates cluster behavior as a decision-making process influenced by interest choices and rational games, incorporating concepts like "Selective Incentives Choice," "Mass Society Theory," "Resource-Mobilization Theory," and "Rational Choice Theory." The structural orientation explores the macro-level relationship between the state, government, and group behavior, building upon Tocqueville’s analysis of the French Revolution. Tocqueville’s argument that the French people engaged in the revolution primarily as a "revolt" rather than to achieve "liberty, equality, and fraternity" serves as a basis for subsequent research investigating concepts such as "Social Change theory," "Polity Model," and "Political Process theory." The cultural orientation focuses on the development of shared beliefs and collective identities within groups, encompassing theories such as "Cultural Marxism theory," "Collective Beliefs theory," "Cultural Constructivism theory," and "Framework Integration theory."

A collective behavior, the strength of one’s identification with a particular group may directly impact their likelihood of engaging in collective actions. On the other hand, group identity can also function as a moderator when exploring the relationship between other variables. In this case, group identity moderates the relationship between an independent variable and a dependent variable. For instance, the effect of social norms or persuasive messages on individual behavior may vary depending on the strength of an individual’s group identity.

**Fig 1:**
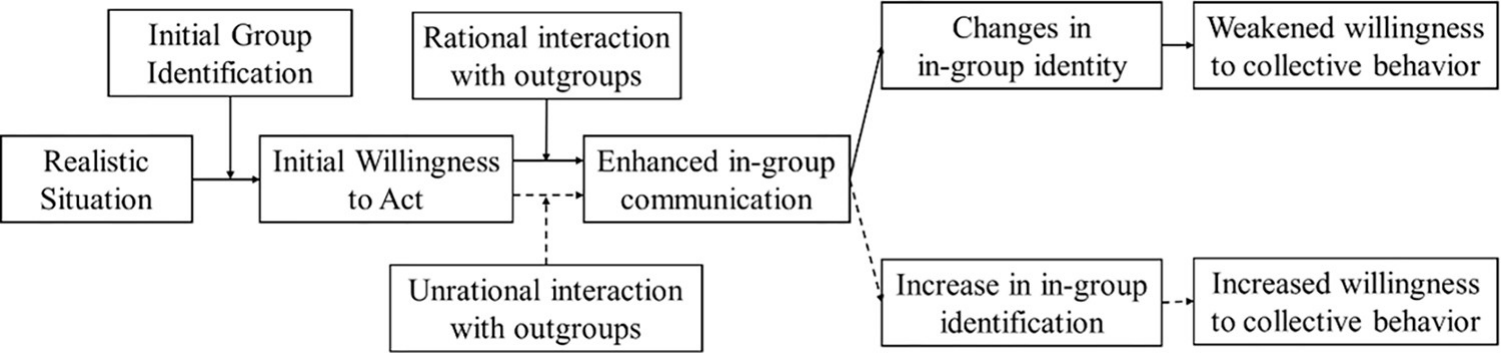
Mechanisms underlying the formation of collective behavior within the framework of the ESIM.

When confronted with a shared reality, the public tends to divide into two factions: those advocating for defending rights through legitimate means, and those becoming disillusioned and distrustful of out-groups, endorsing violent actions. Cooperation between these factions is limited, with moderates initially refraining from rejecting authority figures belonging to out-groups, such as the police. However, the out-groups fail to recognize the diversity within the in-groups and perceive the public as a potentially threatening and unified entity to be controlled. Consequently, the moderates within the in-group shift their stance and adopt a self-defensive position in response to irrational interactions from the out-group, eventually aligning themselves with radical advocates. The out-group’s disregard for the heterogeneity within the in-group justifies and legitimates public resistance and confrontation with the police. This model highlights the significance of intergroup interactions in shaping online collective behavior and provides a dynamic analytical perspective that diverges from the static portrayal of various quantitative models mentioned earlier (Refer to Fig 1).

The model’s predictions were solely based on China-specific data, and further validation using multi-country datasets is required to establish generalizability. Finally, the study did not report iterative refinements of the ANN based on evaluation results, which could potentially improve its predictive power. Given these gaps, this research aims to address the following three questions, taking into account the research case:

Q1: What are the genuine intentions and demands of the Chinese public in response to the "rapid relaxation of epidemic control measures" towards the end of 2022?

Q2: What factors influence the online collective behavior of the Chinese public in this incident, and what is the specific relationship between these factors?

Q3: Can the ESIM effectively explain the online collective behavior exhibited by the Chinese public in this context?

This research is guided by social identity theory and cognitive-behavioral theory, utilizing the ESIM model as the framework. It references various social identity models, including SIMCA, EMSICA, and DDPMAC, and extracts key index elements from each model. These elements include public attention (PA), group negative emotion (NE), group identity (GI), group efficacy (GE), sense of fairness (SF), and intergroup interaction (IG). The study aims to establish a clear understanding of the relationships between these index roles, ultimately constructing a comprehensive theoretical model. This study intends to explore the factors influencing online collective behavior within the framework of the "cognitive-emotional-behavioral" paradigm. The "cognitive" aspect is represented by public attention, while the "emotional" aspect encompasses group negative emotion, sense of fairness, group identity, and group efficacy. The "behavioral" aspect focuses on online collective behavior itself. According to cognitive-behavioral theory, cognition plays a crucial role in coordinating emotional and behavioral changes. Individuals’ inappropriate emotions and behaviors are primarily attributed to irrational cognitive systems. Based on this understanding, the following hypotheses are proposed:

H1: Public attention has a significant effect on group negative emotion, sense of fairness, group identity, and group efficacy.
H1a: Public attention positively influences group negative emotions.
H1b: Public attention negatively affects the sense of justice.
H1c: Public attention positively influences group identity.
H1d: Public attention positively influences group efficacy.

Previous studies have confirmed a significant relationship between group negative emotion and collective behavior. When a group experiences a situation that damages their interests, these negative emotions can effectively stimulate the public’s behavioral intentions. Additionally, a substantial number of scholars suggest that negative group emotions can impact the public’s sense of justice. According to the affect-as-information theory, individuals often rely on their emotional feelings regarding an event as a judgment criterion when making fair judgments. In this context, emotions become reliable information for people to assess fairness. Based on these findings, the following hypotheses are proposed:

H2: Group negative emotions have a significant effect on the sense of justice and online collective behavior.
H2a: Group negative emotion positively affects online collective behavior.
H2b: Group negative emotion negatively influences the sense of justice.

In the field of research exploring the association between perceptions of fairness and online collective behavior, various theories have provided evidence that a diminished sense of fairness can trigger the emergence of online collective behavior. For instance, social identity theory suggests that individuals with a strong sense of group identification are more likely to empathize with negative emotional experiences faced by fellow group members, thus increasing their inclination to engage in collective actions. Building upon these theories, the following hypothesis is proposed:

H3: Sense of justice negatively impacts online collective behavior.

Similarly, in studies focusing on the relationship between group identity and antecedent variables of online collective behavior, significant associations have been discovered between group identity and fairness perception, group efficacy, and online collective behavior. For instance, the SIMCA model suggests that group identity can directly predict online collective behavior and indirectly influence it through perceptions of injustice and group efficacy. Additionally, group identity moderates the effect of group efficacy on behavioral intentions. This moderating effect implies that individuals will consider the costs of participating in collective behavior only when their identification with the group is sufficiently strong. Conversely, individuals with weaker group identification will base their decision to engage in collective behavior more on personal motivations. Based on these findings, the following hypotheses are proposed in this study:

H4: Group identity significantly influences the relationship between perceptions of justice, group efficacy, and online collective behavior.
H4a: Group identity negatively influences the sense of fairness.
H4b: Group identity positively influences group efficacy.
H4c: Group identity positively influences online collective behavior.
H5: Group identity positively moderates the relationship between group efficacy and online collective behavior. In studies examining the relationship between group efficacy and collective behavior, a substantial body of empirical research has demonstrated that group efficacy is a significant predictor of collective behavior. These studies consistently show that the willingness to participate in collective behavior is stronger when the group perceives that the abilities and beliefs of the group can lead to improvements in the situation of its members during certain events. Accordingly, the following hypotheses were formulated in this study:
H6: Group efficacy positively influences online collective behavior within the ESIM framework.

**Fig 2:**
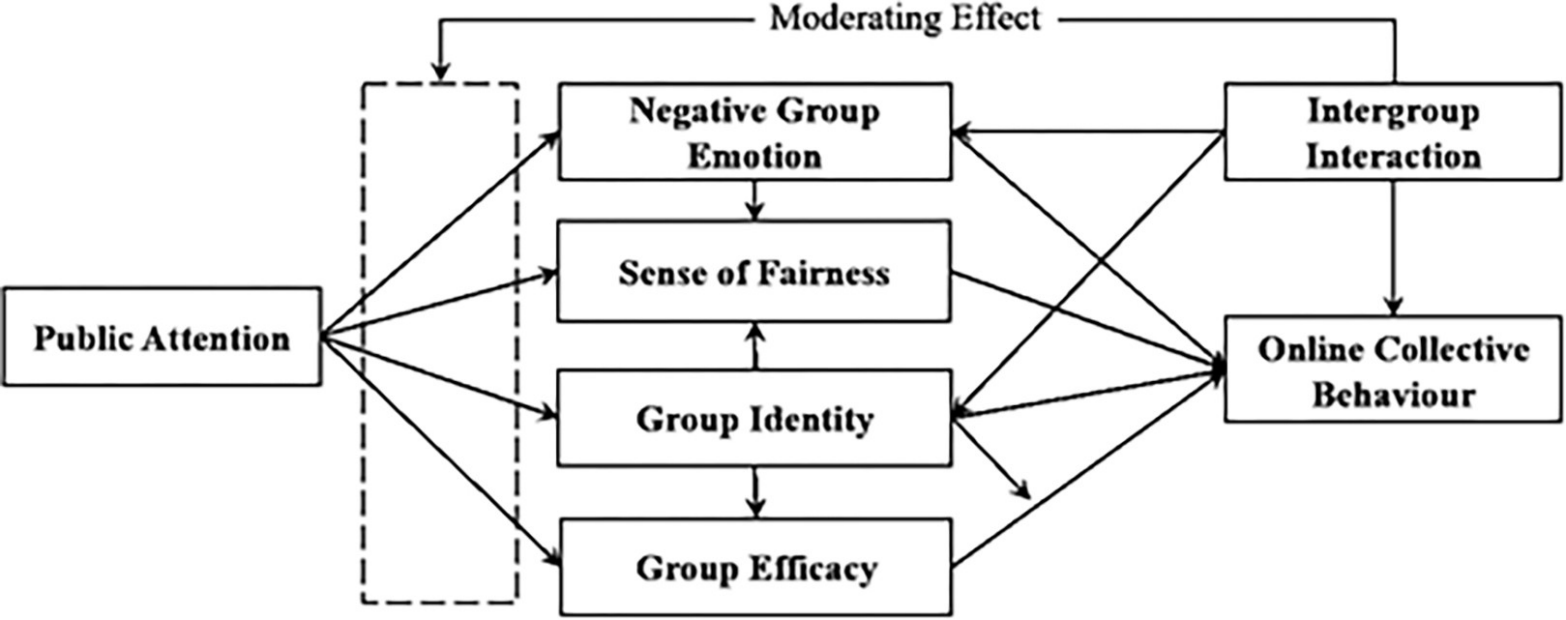
SEM theoretical model diagram for this study.

The model under consideration emphasizes the specific role of intergroup interactions in individuals’ participation in collective behavior [39–44]. Drury and Reicher conducted a study on fan riots, highlighting the dynamics of intergroup interactions and social identity [42, 44]. They suggested that collective behavior often arises due to low-quality interactions between fans and the police, where the police perceive fans as a uniformly threatening group. This perception gradually leads fans to accept the resistance claims of a small number of violent advocates within the group. Subsequent studies have further expanded and deepened these findings. Based on these insights, the following hypotheses were formulated in this research:

H7: Intergroup interaction has a significant effect on group negative emotions, group identity, and online collective behavior.
H7a: The higher the intensity of intergroup interaction, the weaker the group negative emotion.
H7b: The higher the intensity of intergroup interaction, the weaker the group identity.
H7c: The higher the intensity of intergroup interaction, the less likely online collective behavior is to occur.
H8: Intergroup interaction significantly moderates the relationship between the effects of public attention on group negative emotion, sense of justice, group identity, and group efficacy.
H8a: Intergroup interaction significantly moderates the relationship between the effect of public attention on group negative emotion.
H8b: Intergroup interaction significantly moderates the effect of public attention on perceptions of justice.
H8c: Intergroup interaction can significantly modulate the relationship between the effect of public attention on group identity. H8d: Intergroup interaction plays a significant moderating role in the effect of public attention on group efficacy. Drawing upon the aforementioned research assumptions, this study developed a theoretical SEM, as depicted in Fig 2.

The theoretical model of structural equations that was developed reveals a complex relationship between the independent variables and the dependent variable, giving rise to a series of potential mediating paths. These paths will be systematically examined in subsequent analyses to determine their validity in mediating the latent variables [27–32].

### 2.3 Theoretical underpinnings

This study incorporates social identity theory and cognitive-behavioral theory to investigate online collective behavior. Social identity theory emphasizes the significance of group memberships in shaping individuals’ self-perception and their motivation to protect or enhance their social identity. Group identity is considered both as an independent variable influencing cognition, emotion, and behavior, and as a moderator of relationships between other variables. Cognitive-behavioral theory, on the other hand, highlights cognition as the initiator and coordinator of emotional and behavioral changes, with irrational cognitive systems leading to inappropriate emotions and actions. The model employed in this study examines the cognitive dimension through public attention, the emotional dimension through negative emotions, fairness perceptions, group identity, and efficacy, and the behavioral dimension through online collective behavior. It hypothesizes that cognition (public attention) influences emotion and behavior, integrating cognitive, emotional, and behavioral processes in line with cognitive-behavioral theory. This study drew upon social identity theory and cognitive-behavioral theory as its main theoretical foundations. Social identity theory posits that individuals derive a sense of self from their group memberships. It views group identity as both an independent variable influencing cognition, emotion, and behaviors, as well as a moderator of relationships between other variables. By emphasizing the importance of group memberships in shaping self-concept and motivation to protect social identity, social identity theory provided justification for including group identity as a key construct in the model. Cognitive-behavioral theory highlights the role of cognition in coordinating emotional and behavioral changes. It proposes that irrational cognitive systems lead to inappropriate emotions and behaviors. This theory lent support to the inclusion of the "cognitive" dimension represented by public attention in the model. It justified examining how cognition influences emotions and behaviors by hypothesizing that public attention would impact group emotions and behavior through cognitions about the issues. In addition, the study drew upon the ESIM of collective action to develop a theoretical framework for analyzing online collective behavior. ESIM views collective behavior as resulting from interactions between social identities and intergroup dynamics. It synthesizes perspectives on collective identity, fairness perceptions, emotions, and efficacies as antecedents impacting participation. ESIM provided theoretical rationale for incorporating these constructs in the model and proposing relationships between them based on its conceptualizations. For example, it justified hypothesizing that group identity would directly influence behaviors and indirectly impact them through fairness and efficacy perceptions based on its view of identity as shaping attitudes. ESIM also supported examining the moderating role of identity by treating it as a moderator per its conceptualization. By grounding the study in these three theories, the theoretical underpinnings section more comprehensively and critically discusses how the theories lend support to the model, hypotheses, and variable relationships proposed. The integration of social identity theory, cognitive-behavioral theory, and ESIM provides a robust theoretical basis for the study.

## 3. Research Methodology

### 3.1 Data collection and procedures and survey sample

This study focused on Chinese Internet users residing inland and employed a comprehensive and random placement strategy for the questionnaire across various social media platforms, news and information platforms, and search engine platforms.

To collect the data, a multi-stage approach was utilized. First, prominent universities across China were identified with active researchers and academics in the fields related to the scope of this study. Professors and researchers from the selected universities comprised the initial contact pool. They were contacted through email with an invitation to participate in an anonymous online survey regarding the research topic. Snowball sampling was then utilized by asking these initial contacts to recommend other potential participants from their professional networks. In this study, the data used for analysis was sourced from dataset available as supplementary materials. Prior to data collection, permission was obtained from the data source to access and utilize the dataset for research purposes. The terms and conditions set forth by the data source were carefully reviewed and adhered to throughout the data collection and analysis process. To ensure compliance with the terms and conditions, we implemented stringent data handling procedures. These included maintaining data confidentiality, using secure storage systems, and strictly adhering to data protection regulations. Simultaneously, common online survey distribution platforms were leveraged to directly target relevant industry professionals, government officials, and members of the general public. Survey links were shared on professional online forums and social media groups related to the research focus areas to expand the reach of data collection. To obtain a geographically diverse sample, distribution was conducted nationwide across different Chinese provinces and municipalities through regional social media groups and online communities. To achieve better response rates, tailored outreach was conducted in multiple formats including phone calls, video conference introductions, and face-to-face discussion sessions with key informants. Participation was entirely voluntary and anonymous to ensure participants’ comfort in openly sharing their professional insights and lived experiences. A minimum professional experience of 3 years in related domains was required for industry experts and policymakers surveyed. A total of 1,769 responses were collected, and after removing 143 invalid responses, the valid response rate was 91.92%. Regarding public perspectives on epidemic prevention and control, 65.69% of respondents tended to favor a gradual relaxation of restrictions over time, while only 34.32% supported a swift lifting of the blockade, indicating a nearly twofold difference. The male population accounted for 60.40% of the sample, while females accounted for 39.61%. Urban households comprised 64.95% of the total, while rural households accounted for 35.06%. Additionally, close to 60% of respondents had tertiary education qualifications, indicating a higher level of education. The majority of survey participants fell within the age range of 20–49, which is the most active age group for Internet usage in inland China. In terms of income distribution, based on the criteria outlined in the China Statistical Yearbook 2021, the very-low-income group constituted 8.46% of the sample, the low-income group accounted for 42.44%, the middle-income group comprised 41.76%, and the high-income group represented 6.87%. Concerning subjective class identity, 14.64% of respondents identified themselves as belonging to the upper class, 27.12% to the upper-middle class, 40.78% to the middle class, 13.47% to the lower-middle class, and 3.9% to the lower class. The characteristics of the study sample closely align with those of inland Internet users, as reported in the China Internet Development Statistics Report. In addition, the mean values of the dimensions in the questionnaire are relatively balanced (M_PA_ = 3.83, SD_PA_ = 0.94; M_IN_ = 4.97, SD_IN_ = 1.35; M_GI_ = 3.78, SD_GI_ = 0.92; M_IG_ = 5.13, SD_IG_ = 1.51; M_SF_ = 5.23, SD_SF_ = 1.45; M_GE_ = 3.72, SD_GE_ = 1.51; M_NE_ = 3.77, SD_NE_ = 1.07), with small variations in standard deviation and low dispersion.

### 3.2 Data analysis

#### 3.2.1 Reliability and validity tests

In this formal research, the reliability of the questionnaire was assessed using Cronbach’s alpha coefficient method. The calculated Cronbach’s alpha coefficients for each dimension were found to be higher than 0.8 (PA = 0.88, IN = 0.90, GI = 0.91, IG = 0.91, SF = 0.95, GE = 0.81, and NE = 0.89), indicating a good level of reliability for the questionnaire. The validity testing focused on structural validity, convergent validity, and discriminant validity. The results of the validation factor analysis demonstrated that all indicators met the fit requirements (GFI = 0.903>0.9, AGFI = 0.889>0.8, NFI = 0.928>0.9, RFI = 0.921>0.9, IFI = 0.954>0.9, TLI = 0.950>0.9, CFI = 0.954>0.9, PNFI = 0.850>0.5, PCFI = 0.874>0.5, p<0.001, CMIN/DF = 2.623<3, RMSEA = 0.040<0.05), indicating satisfactory structural validity of the scale. Table 1 shows the reliability and convergent validity of the variable combinations examined in the study. Reliability refers to the consistency and stability of the measurements, while convergent validity assesses the degree to which items within a variable combination measure the same underlying construct.

**Table 1:** Reliability and convergent validity of variable combinations.

Latent Variable	p	CR	AVE
PA	0.000	0.9695	0.9409
SF	0.000	0.989	0.9781
GI	0.000	0.9251	0.8606
GE	0.000	0.8122	0.5904
IG	0.000	0.9069	0.7094
IN	0.000	0.9044	0.6548
NE	0.000	0.872	0.6301

*p<0.05

**p<0.01

***p < 0.001

The subsequent studies revealed strong convergent validity for the scale, as all seven latent variables exhibited CR values exceeding 0.6 and AVE values exceeding 0.5 (p <0.001). The lowest square root of AVE value was 0.768, which exceeded the highest correlation coefficient value of 0.652. These results indicate a high level of discriminant validity for the scale. Additionally, significant correlations were observed among the seven latent variables at different levels (p<0.05). Table 2 presents the results of the validity assessment for the variables included in the study. Convergent validity, which indicates the extent to which items within each latent variable are measuring the same underlying construct, was evaluated using two indicators: the composite reliability (CR) and the average variance extracted (AVE).

**Table 2:** Validity of variables.

	PA	SF	IG	NE	GE	GI	IN
PA							
SF	0.378***						
IG	0.373**						
NE	0.556*	0.507*	0.402***				
GE	0.485**	0.652**	0.506*	0.592*			
GI	0.584*	0.478*	-0.485**	0.582*	0.557**		
IN	0.498*	0.365**	-0.318**	0.437**	0.391**	0.396**	
AVE square root absolute value	0.970**	0.989**	0.842**	0.794***	0.768***	0.928*	0.809***

*p<0.05

**p<0.01

***p<0.001

#### 3.2.2 Research instruments

The concept of "need for orientation" provides a robust psychological framework for measuring public agenda attention. Recent studies [33–38] have primarily focused on components such as personal participation, personal concern, and personal interest. Taking inspiration from Yang Yu and Lee’s measurement tool, this study assessed the public’s attention to the event of lifting the epidemic restrictions by considering both issue awareness and involvement. In this study, the measurement of negative group emotion involved presenting participants with a situational scenario and encouraging them to identify with both the situation and their respective group. The Positive and Negative Affect Scale (PANAS) developed by David Watson in 1988 served as a reference for selecting appropriate measurement words such as "fearful, scared, nervous, alert, fidgety, irritable" [39–41]. In the field of communication, it is common to assess individuals’ "intention for online collective behavior" rather than the actual occurrence of such behavior. This study adopted a measurement approach utilized by previous scholars. To measure participants’ intention for online collective behavior, we developed appropriate materials based on an in-depth article titled "How the Aging Countryside Reads Through the Winter of the Epidemic." Five questions were formulated to gauge participants’ inclination to engage in online collective behavior. All the variables, including intention for online collective behavior, were measured using a Likert scale with a range of 5 to 7 points. Additionally, demographic variables such as gender, age, household category, education level, monthly income level, and subjective class identity were examined. The subjective class identity scale employed in this study was adapted from the MacArthur Scale of subjective class and consisted of a five-level classification that took into account the social context of mainland China. In order to ensure a representative sample, this research specifically targeted Chinese Internet users residing in inland regions. To achieve this, a comprehensive and random placement strategy was implemented across various social media platforms, news/information platforms, and search platforms. Out of the total initial responses received, 1,626 valid questionnaires were analyzed. The characteristics of the sample closely corresponded to those reported in government statistics for Chinese Internet users in inland regions. The sample encompassed individuals of both genders aged 19 years and above, individuals with urban or rural registrations, and individuals with diverse education levels and incomes that were representative of the population residing in inland regions. To capture a diverse range of perspectives, the study employed proportional quotas based on demographic factors. The purpose of this sampling strategy was to comprehensively capture the viewpoints of Chinese Internet users residing in inland regions. Although the sample may not have been a perfect match, it did reflect the key characteristics of the target population. By transparently describing the sampling procedures and demonstrating the alignment with population parameters, the credibility and generalizability of the findings to the target population are strengthened.

The study utilized a multi-stage sampling method to obtain a representative sample of Chinese Internet users residing in mainland China. Firstly, all provinces and autonomous regions were stratified based on their geographic location and population distribution. Secondly, three provinces were randomly selected from each region using a random number generator. Thirdly, within the selected provinces, urban and rural districts/counties were further stratified. Two districts/counties from urban areas and one from rural areas were randomly chosen in each province. Fourthly, the target population consisted of individuals aged 19 years or older within the selected districts/counties. Quotas were established to ensure a proportional representation of gender, age, education level, occupation, and income that aligned with the population parameters. Finally, an online random number table was utilized to select potential respondents by matching the established quotas from user databases of popular social platforms, search histories, and mobile application data in the respective areas. By employing a multi-stage stratified random sampling approach and setting quotas to match population distributions, the study aimed to obtain a sample that accurately represented Chinese Internet users residing in mainland China.

## 4. Results

### 4.1 Difference tests

This study examined the impact of demographic variables on attitudes towards epidemic prevention and control, as well as the influence of public attention, intergroup interaction, group identity, group efficacy, negative group emotions, online clustering behavior, and sense of fairness. The variability of demographic variables was assessed using the chi-square test, while the variability of the aforementioned factors was analyzed using independent samples t-tests and one-way ANOVA tests. The results yielded several significant research findings, which are elaborated upon as follows:

Regarding attitudes towards epidemic control, several significant findings emerged. Firstly, there was a gender difference, as males showed a higher inclination towards supporting rapid unblocking compared to females (χ2 = 181.103a, p<0.001). Secondly, urban household members were more supportive of rapid unblocking compared to their rural counterparts (χ2 = 87.832a, p<0.001). Thirdly, individuals with lower educational attainment demonstrated a greater propensity for supporting rapid unblocking compared to those with higher education levels (χ2 = 64.301a, p<0.001). Fourthly, in terms of income, both the objective income and subjective class division of the public, individuals in the higher income bracket exhibited a stronger preference for rapid unblocking compared to those in the lower-middle income bracket (χ2 subjective = 128.012a, χ2 objective = 107.336a, p<0.001). Regarding public attention, several significant findings emerged. Firstly, younger individuals exhibited higher levels of attention and involvement compared to older individuals (F = 5.832, p<0.001). Secondly, individuals with higher levels of education demonstrated stronger attention and higher involvement than those with lower levels of education (F = 7.005, p<0.001). Thirdly, irrespective of objective or subjective class distinctions, individuals in the high-income stratum displayed stronger attention and higher involvement compared to those in the lower-middle income stratum (F objective = 2.803, p = 0.039; F subjective = 6.061, p = 0.039). In terms of intergroup interaction, noteworthy findings were observed. Firstly, individuals with lower levels of education displayed greater attention to intergroup interaction and engaged in more frequent and higher-quality interactions with outgroups compared to individuals with higher levels of education (F = 6.263, p<0.001). Secondly, regardless of objective or subjective class identity, individuals in the middle and high-income strata exhibited stronger attention to the reasonableness of intergroup interaction and engaged in frequent and high-quality interactions with outgroups compared to individuals in the low-income stratum (F objective class = 4.699, p<0.001; F subjective = 18.343, p<0.001).

Concerning group negative emotions, significant findings were observed. Firstly, males displayed stronger negative emotions than females (T = 3.572, p<0.001). Secondly, individuals with lower levels of education exhibited stronger negative emotions compared to those with higher levels of education (F = 5.269, p<0.001). Thirdly, regarding class distinctions, whether based on objective or subjective strata, individuals in the middle-upper, upper-middle, and high-level strata demonstrated stronger negative emotions compared to individuals in the middle-lower and lower-level strata (F objective = 6.482, p<0.001; F subjective = 13.897, p<0.001). Regarding the sense of justice, several noteworthy findings were observed. Firstly, individuals with a master’s degree or higher education exhibited the weakest sense of justice (F = 10.102, p<0.001). Secondly, regardless of objective or subjective class identity, individuals in the middle and high-income strata displayed a stronger sense of justice compared to those in the low-income strata (F objective = 3.604, p<0.001; F subjective = 21.170, p<0.001).

### 4.2 Model fitting

The model was constructed using AMOS 25.0, with measurement terms imported and the maximum likelihood method employed. The fit of the model was evaluated using various fit indices. The results indicated that the model fit the data well, as evidenced by the following fit indices: CMIN/DF = 2.798<3.0; GFI = 0.902>0.9; AGFI = 0.888>0.8; IFI = 0.950>0.9; TLI = 0.946>0.9; CFI = 0.950>0.9; RFI = 0.918>0.9; NFI = 0.924>0.9; PNFI = 0.850>0.5; PCFI = 0.874>0.5; RMSEA = 0.042<0.05. These indicators all met the criteria for good fit, suggesting that the measurement model exhibited a high level of fit overall.

Based on the path coefficients presented in the Table 3, it is evident that, with the exception of the PA→SF and IG→NE paths, which are not statistically significant (p>0.05), the coefficients for the remaining paths reached a significant level (p<0.05). Consequently, hypotheses H1b and H7c do not hold. Specifically, intergroup interaction (IG) exhibits a significant negative impact on group negative emotion (IN) (p<0.001), confirming hypothesis H7a. Public attention (PA) demonstrates a significant positive effect (p<0.001) on group negative emotion (IN), validating hypothesis H1a. Intergroup interaction (IG) exerts a significant negative effect (p<0.001) on group identity (GI), supporting hypothesis H7b. Public attention (PA) has a significant positive effect (p<0.001) on group identity (GI), confirming hypothesis H7b. Both group identification (IG) and public attention (PA) significantly contribute (p<0.001) to group efficacy (GE), validating hypotheses H4b and H1d. Group negative emotion (IN) and group identity (GI) significantly influence (p<0.001) the sense of fairness (SF), rendering hypotheses H2b and H4a invalid. Group identity (GI), group efficacy (GE), and negative group emotion (IN) have significant positive effects (p<0.001) on online collective behavior (NE), thereby confirming hypotheses H4c, H6, and H2a.

**Table 3:** Path coefficients for SEM.

Pathway	Unstandardized Coefficient	Standardized Coefficient	p
IG→IN	-0.172	-0.169	***
PA→IN	0.75	0.431	***
PA→GI	0.49	0.502	***
IG→GI	-0.204	-0.356	***
PA→SF	-0.022	-0.013	0.816
PA→GE	0.181	0.171	**
GI→GE	0.633	0.584	***
GI→SF	0.894	0.513	***
IN→SF	0.189	0.193	***
GI→NE	0.459	0.379	***
IG→NE	-0.017	-0.025	0.494
GE→NE	0.243	0.217	***
IN→NE	0.121	0.178	***
SF→NE	0.084	0.12	**

*p<0.05

**p<0.01

*** p<0.001

**Fig 3:**
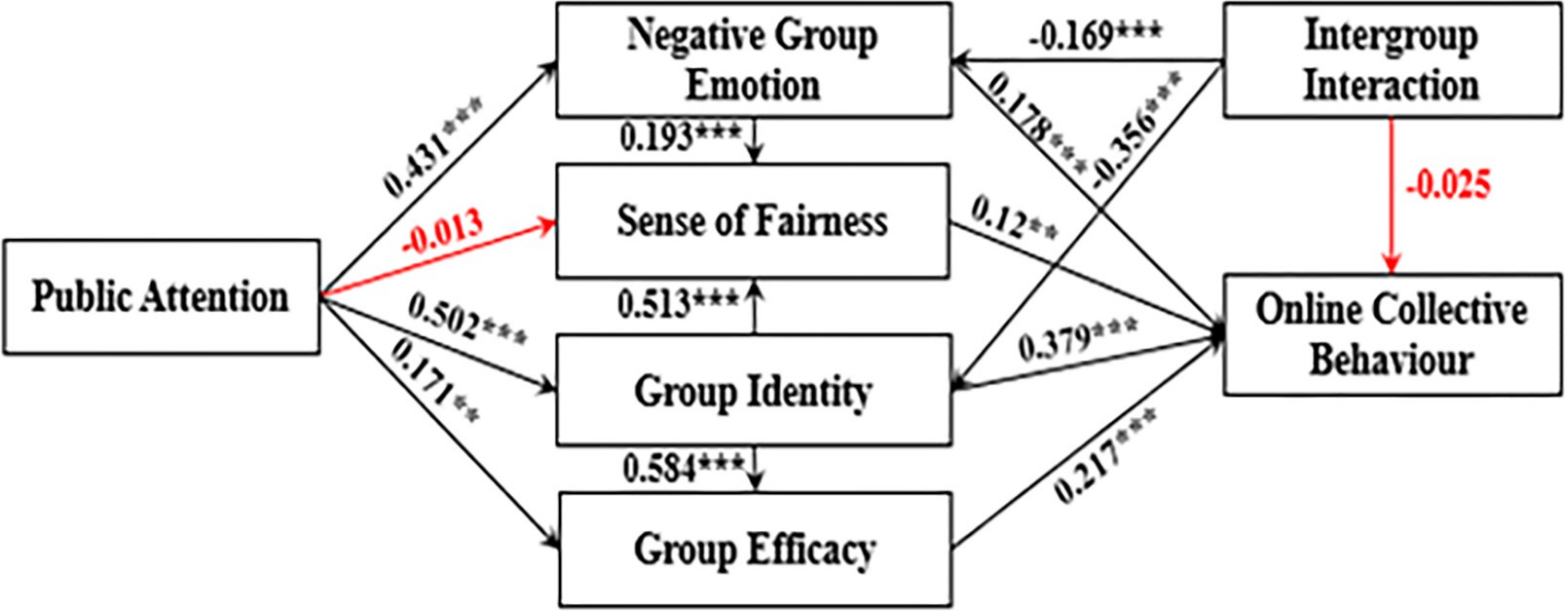
Model diagram of the actual structural equations.

The theoretical model is largely supported. However, there are two paths with insignificant effects and two paths that contradict the original hypotheses in the actual model, necessitating further discussion and explanation. The actual model diagram is depicted in Fig 3, with the reddish bold line indicating an insignificant path.

### 4.3 Mediating effect of latent variables tests

After confirming the satisfactory fit of the model, the present study assessed the latent variable mediation effect using AMOS 25.0. This analysis employed Bootstrap repeated sampling with 5000 samples and determined the mediated effects based on confidence intervals at a 95% confidence level. The specific mediated effects are presented in Table 4.

**Table 4:** Standardized coefficient of latent variable mediation effect.

Mediator variable path	Efficacy value	Bias-corrected 95%CI		
		Lower	Upper	p
PA→IN→NE	0.078***	0.040	0.129	0.000
PA→IN→SF→NE	0.010*	0.002	0.023	0.021
PA→SF→NE	0.001	-0.026	0.029	0.928
PA→GI→NE	0.182**	0.085	0.391	0.001
PA→GI→SF→NE	0.030*	0.006	0.066	0.025
PA→GI→GE→NE	0.062*	0.012	0.123	0.032
PA→GE→NE	0.040	-0.010	0.131	0.131
IG→IN→SF→NE	0.004*	0.001	0.011	0.017
IG→GI→NE	0.133**	0.066	0.286	0.001
IG→GI→SF→NE	0.022*	0.005	0.048	0.023
IG→GI→GE→NE	0.046*	0.011	0.098	0.028
PA→IN→SF	0.083***	0.044	0.128	0.000
IN→SF→NE	0.023*	0.004	0.051	0.021
PA→GI→SF	0.246***	0.159	0.532	0.000
GI→SF→NE	0.062*	0.011	0.123	0.026
PA→GI→GE	0.282***	0.184	0.559	0.000
GI→GE→NE	0.127*	0.021	0.238	0.034
IG→IN→SF	0.031***	0.011	0.059	0.000
IG→GI→SF	0.181**	0.093	0.346	0.001
IG→GI→GE	0.207**	0.108	0.373	0.001
IG→IN→NE	0.029***	0.013	0.055	0.000

*p<0.05

**p<0.01

*** p<0.001

### 4.4 Moderating effect of latent variables tests

**Fig 4:**
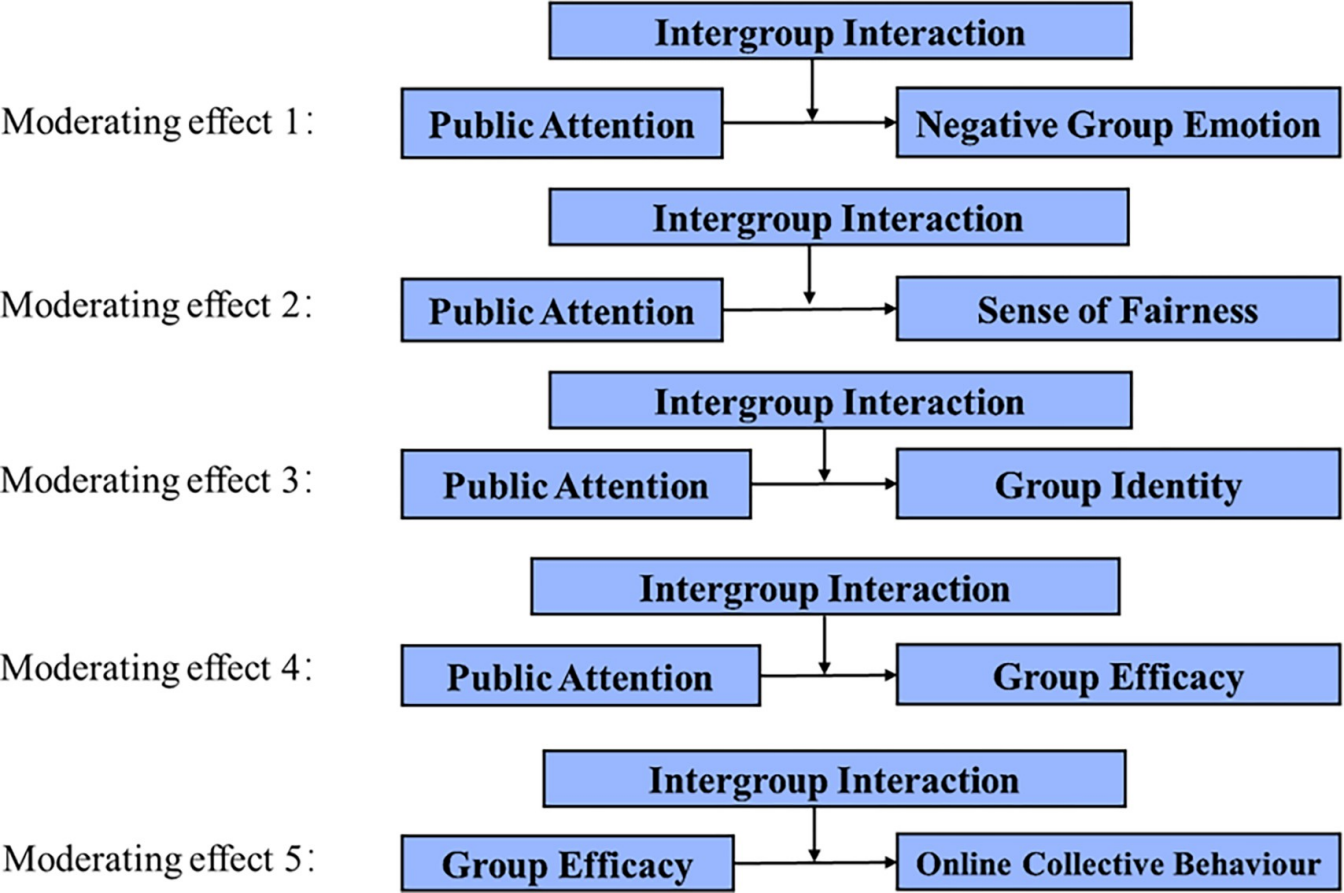
Path diagram of the moderating effects of latent variables.

The theoretical model in this study includes five sets of paths to test for moderating effects, as depicted in Fig 4. To assess the latent variable moderating effects, it is necessary to determine the factor loadings of the independent and moderating variables separately. The observed variables with high factor loadings are prioritized. Additionally, items that exhibit high loadings on the independent variable are matched with items that have high loadings on the moderator variable, while items with low loadings are matched with items that have low loadings on the moderator. Subsequently, latent variable interaction effect models were constructed and evaluated for fit.

Upon analysis, the p-values for moderating effects 1–4 were found to be statistically insignificant (p>0.05). Consequently, the moderating effects of intergroup interactions between public attention and group identity, negative group emotions, sense of fairness, and group efficacy were not supported, leading to the rejection of hypothesis H8 and its sub-hypotheses (H8a, H8b, H8c, and H8d). Conversely, the p-value of moderating effect 5 was less than 0.001, indicating a highly significant moderating path between group identity, group efficacy, and online collective behavior, thereby validating hypothesis H5. As displayed in Table 5, the value of the high grouping moderating effect is 0.666, with a confidence interval of (0.511, 0.833), and p = 0.000<0.001, signifying the significant nature of the high grouping moderating effect. Similarly, the value of the low grouping moderating effect is 0.266, with a confidence interval of (0.099, 0.42), and p = 0.003<0.01, indicating the significance of the low grouping moderating effect as well.

**Table 5:** High-low score moderating effects of group identification.

group	effect	Bias-corrected 95%CI			Percentile 95%CI		
		Lower	Upper	P	Lower	Upper	P
high	0.666	0.511	0.833	0.000	0.512	0.835	0.000***
mean	0.466	0.345	0.593	0.000	0.343	0.591	0.000***
low	0.266	0.099	0.42	0.003	0.097	0.418	0.003**
std_xm	0.182	0.102	0.292	0.000	0.101	0.291	0.000**
std_high	0.607	0.47	0.743	0.000	0.473	0.747	0.000***
std_mean	0.424	0.321	0.526	0.000	0.319	0.524	0.000***
std_low	0.242	0.091	0.374	0.003	0.089	0.372	0.003**

*p<0.05

**p<0.01

*** p<0.001

**Fig 5:**
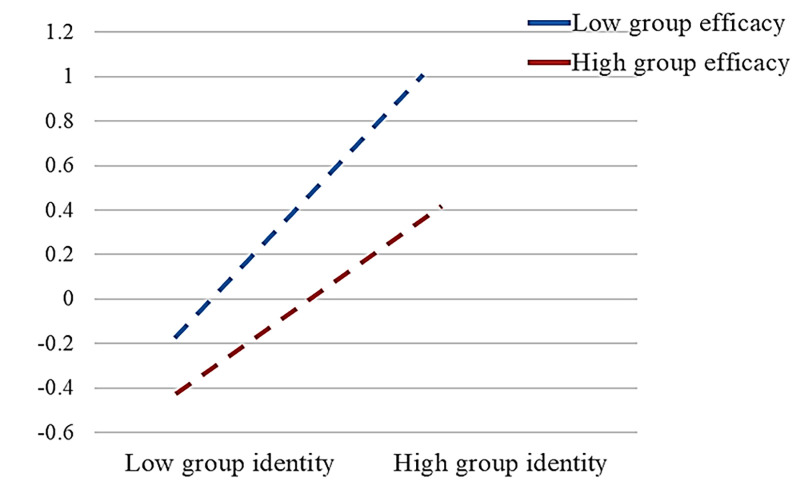
Decomposition of group identity moderating effects.

Based on the decomposition of the moderating effects presented in Fig 5, it is evident that there is an intuitive observation that higher levels of public in-group identity amplify the strength of the moderating effect of public group efficacy on online collective behavior.

This study shows the multifaceted considerations involved in evaluating the efficacy and environmental consequences of waste disposal approaches. As a result, it serves as a valuable resource for researchers, policymakers, and waste management professionals, aiding in the comprehension and resolution of sustainability issues pertaining to waste management. Results establishes a framework for examining the environmental, economic, and social aspects of waste disposal, thereby facilitating well-informed decision-making and the formulation of effective strategies to mitigate the adverse environmental and human health impacts associated with plastic and mixed waste.

## 5. Discussions

### 5.1 Social justice and people-centralism: Visions and demands behind differential political trust

Based on the research findings of this study, it is evident that the average value of systemic sense of justice is 5.18, while the average value of self-justice is 5.16. The paired samples t-test results indicate that there is no significant difference between these two measures (p = 0.216>0.05). On the other hand, the mean value of overall sense of justice is 5.23, and the one-sample mean test reveals a significant result (p = 0.000<0.001). This implies that during the period of epidemic prevention and control, both the public’s evaluation of fairness in the state system and their perception of fairness in personal experiences skewed positively. These results suggest that the majority of the public possessed a strong sense of fairness during the epidemic prevention and control measures, indicating a substantial level of trust in the governing capacity of the Communist Party of China (CPC) and the government’s commitment to serving the public. Contrary to the original hypothesis, the two paths in the model, namely "negative group sentiment → sense of fairness" and "group identity → sense of fairness," exhibit opposite trends [42–48]. This discrepancy may be attributed to the weak negative relationship between the negative emotions and in-group identities of the public groups that support the gradual liberalization of epidemic control. Additionally, there might be other influential variables that have a more significant impact on the perception of fairness. The significant positive effect observed in the "sense of fairness → online collective behavior" path can be explained by the deeply rooted trust the public holds in the CPC and the government [49–52]. Stott et al. [53] discovered that when outgroups listen to "public opinion" in a "low profile" manner, characterized by higher quality intergroup interactions and more harmonious intergroup relations, the likelihood of intergroup conflict significantly decreases. Many works investigate various topics, including the impacts of live chat on service-product purchase, institutional investor ESG activism, and the role of natural resources and green innovations in the Chinese economy [54–58]. Additionally, these studies examine the future of green energy and renewable resources. They also analyze individual investors’ trading behavior and gender differences, as well as the performance evaluation of environmental non-governmental organizations [59–63]. Furthermore, the studies focus on opinion formation analysis, violence against healthcare workers, and nursing students’ risk perception during the COVID-19 pandemic [64–68]. They also explore group membership and behavior modulation, the changing dynamics of China’s digital trade, the spatiotemporal evolution of global innovation networks, and the spatial network structure of ethnic regions in Northwest China [69, 70]. Collectively, these studies provide valuable evidence and insights into various aspects of business, economic, social sciences, public health, and neuroscience.

### 5.2 Theoretical implications and practical implications

The study’s findings carry substantial theoretical and practical implications. Theoretically, the study validates the applicability of the ESIM in explaining online collective behavior within the Chinese context, thereby extending its scope beyond Western societies. Moreover, the study provides support for the model’s capacity to encompass both intense and moderate forms of collective action, demonstrating its versatility across a spectrum of intensities. Additionally, the study highlights the moderating role of group identity and intergroup interactions, shedding light on previously unexplored mechanisms within the ESIM framework. Furthermore, the investigation establishes positive intergroup relations and rational claims as catalysts for tempered resistance behaviors online, facilitating a nuanced comprehension of the factors that shape such behaviors. In practical terms, the study offers guidance to policymakers regarding the distinguishing features of online collective actions and the elements that influence these behaviors during policy shifts. Specifically, the research underscores the perception of vulnerability among the middle class, suggesting the need to consider socioeconomic impacts when implementing changes. Furthermore, the study indicates that fairness perceptions and established trust in authorities influence moderate behaviors, thereby assuring policymakers of public cooperation during transitional periods. The study also underscores the significance of fostering positive intergroup interactions in order to promote rational online engagement and cooperation with authorities. Lastly, the study serves as a point of reference for cultivating inclusive identities and moderating intergroup relations, thereby facilitating constructive and secure online discussions. These practical implications provide valuable insights for policymakers and stakeholders involved in the management of online collective behavior.

In the theoretical model, the mediation effects of most paths are supported, except for two paths that do not yield significant results. These non-significant paths are "public attention → sense of fairness → online collective behavior" and "public attention → group effectiveness → online collective behavior." Further analysis and discussion are required to investigate these findings. Regarding the relationship between public attention and online collective behavior, both group negative emotion and group identity can mediate the influence of public attention. However, the sense of fairness and group efficacy do not mediate this relationship. Nonetheless, the sense of fairness can mediate the paths of "negative group emotion → online collective behavior" and "group identity → online collective behavior." Notably, the chain reactions of "public attention → group negative emotion → sense of fairness → online collective behavior" and "public attention → group identity → sense of fairness → online collective behavior" are statistically significant, with effect ratios of 9.43% and 9.74% respectively. Group efficacy, on the other hand, can be mediated by the path "group identity → online collective behavior," resulting in the significant chain reaction of "public attention → group identity → group efficacy → online collective behavior" with an effect ratio of 15.16%. In relation to the association between intergroup interaction and online collective behavior, both group identity and negative group emotions can directly mediate this relationship, while the sense of fairness and group efficacy cannot. However, the sense of fairness can mediate the paths of "negative group emotions → online collective behavior" and "group identity → online collective behavior." As a result, the chain reactions of "intergroup interaction → group negative emotion → sense of fairness → online collective behavior" and "intergroup interaction → group identity → sense of fairness → online collective behavior" are statistically significant, with effect ratios of 7.41% and 9.05% respectively. Group efficacy, on the other hand, can be mediated by the path "group identity → online collective behavior," resulting in the significant chain reaction of "intergroup interaction → group identity → group efficacy → online collective behavior" with an effect ratio of 13.77%.

## 6. Conclusion

The study explored online collective behavior in response to COVID-19 lockdown relaxation in China, employing surveys and the ESIM. The findings revealed rational resistance characterized by moderated negotiation calls, aligning with the ESIM framework within the Chinese context. The study validated ESIM’s applicability beyond Western societies, showcased its versatility across intensity levels, and highlighted the moderating roles of group identity and intergroup dynamics. Practical implications suggested policymakers consider socioeconomic impacts, maintain positive intergroup interactions, and leverage fairness perceptions and trust to ensure public cooperation. Limitations included limited data diversity and variables, recommending the incorporation of longitudinal data from multiple sources and qualitative insights through interviews. Future research recommendations included validating predictions using multi-country datasets and iteratively refining models. In conclusion, the study shows valuable insights for promoting constructive online participation and understanding online collective behavior in developing societies, benefiting authorities and community managers.

## 7. Limitations and future research

The study investigates the online collective behavior of the Chinese population. Despite attempts to achieve sample balance, there are limitations in the distribution of the sample, such as the absence of data from Internet users aged 60 and above and the public in Northwest China. The research primarily employs a quantitative approach but lacks a comprehensive analysis of qualitative research methods. The ESIM model, originally developed by British scholars within the context of the UK’s political landscape, has predominantly been utilized by European and American researchers to explain collective behavior in their respective countries. This research represents an initial effort to apply the ESIM model to mainland China, opening up several important avenues for further inquiry. Future researchers can examine the explanatory validity of the ESIM model in countries with socialist regimes other than China and assess whether the model’s explanatory power differs across diverse political systems. Moreover, it is crucial to identify the specific factors contributing to the lack of explanatory validity and comprehend why these factors hinder its effectiveness. Exploring these research questions holds substantial value and warrants further investigation.

## Supporting information

S1 Dataset The dataset employed to present the results in Tables 6 to 18 within the main body of this study. (DOCX)

S1 File The dataset employed to present the results in Tables 6 to 12 within the main body of this study. (DOCX)

S2 File The dataset employed to present the results in Tables 13 to 18 within the main body of this study. (DOCX)
