# Supplementary material for: Rational resistance amidst gentle breeze and mild rain: Examining online collective behavior among the Chinese public using the elaborated social identity model
Source: PLoS One. 2024 May 24;19(5):e0303603. doi: 10.1371/journal.pone.0303603 (PMC11125461; doi:10.1371/journal.pone.0303603)
Supplement: S1 Dataset — (DOCX) [file pone.0303603.s001.docx]

**DATA AND TABLES**

**Table 6:** Reliability Analysis of Variables

| **Variables** | **items** | **CITC**** | **Item deleted α** | **Cronbach α**** |
| --- | --- | --- | --- | --- |
| PA | PA1 | 0.764 | 0.907 | 0.920 |
|  | PA2 | 0.735 | 0.910 |  |
|  | PA3 | 0.745 | 0.909 |  |
|  | PA4 | 0.722 | 0.911 |  |
|  | PA5 | 0.812 | 0.902 |  |
|  | PA6 | 0.667 | 0.916 |  |
|  | PA7 | 0.824 | 0.900 |  |
| IN | IN1 | 0.708 | 0.850 | 0.876 |
|  | IN2 | 0.654 | 0.860 |  |
|  | IN3 | 0.658 | 0.859 |  |
|  | IN4 | 0.648 | 0.860 |  |
|  | IN5 | 0.752 | 0.842 |  |
|  | IN6 | 0.670 | 0.857 |  |
| IG | IG1 | 0.818 | 0.907 | 0.926 |
|  | IG2 | 0.851 | 0.896 |  |
|  | IG3 | 0.846 | 0.898 |  |
|  | IG4 | 0.803 | 0.913 |  |
| GI | GI1 | 0.661 | 0.888 | 0.899 |
|  | GI2 | 0.569 | 0.894 |  |
|  | GI3 | 0.735 | 0.882 |  |
|  | GI4 | 0.638 | 0.890 |  |
|  | GI5 | 0.707 | 0.884 |  |
|  | GI6 | 0.674 | 0.888 |  |
|  | GI7 | 0.596 | 0.892 |  |
|  | GI8 | 0.769 | 0.879 |  |
|  | GI9 | 0.653 | 0.889 |  |
| SF | SF1 | 0.699 | 0.899 | 0.910 |
|  | SF2 | 0.603 | 0.904 |  |
|  | SF3 | 0.736 | 0.897 |  |
|  | SF4 | 0.691 | 0.900 |  |
|  | SF5 | 0.735 | 0.897 |  |
|  | SF6 | 0.588 | 0.905 |  |
|  | SF7 | 0.654 | 0.902 |  |
|  | SF8 | 0.522 | 0.908 |  |
|  | SF9 | 0.693 | 0.900 |  |
|  | SF10 | 0.642 | 0.903 |  |
|  | SF11 | 0.666 | 0.901 |  |
| GE | GE1 | 0.752 | 0.792 | 0.861 |
|  | GE2 | 0.667 | 0.867 |  |
|  | GE3 | 0.802 | 0.742 |  |
| NE | NE1 | 0.731 | 0.873 | 0.894 |
|  | NE2 | 0.665 | 0.887 |  |
|  | NE3 | 0.826 | 0.850 |  |
|  | NE4 | 0.735 | 0.872 |  |
|  | NE5 | 0.749 | 0.869 |  |

Table 7: Reliability statistics table of each variable

| **Variables** | **Cronbach α** | **Cronbach's Alpha based on standardized terms** | **Number of items** |
| --- | --- | --- | --- |
| PA | 0.876 | 0.877 | 7 |
| IN | 0.897 | 0.896 | 6 |
| GI | 0.907 | 0.908 | 9 |
| IG | 0.906 | 0.906 | 4 |
| SF | 0.953 | 0.953 | 11 |
| GE | 0.810 | 0.812 | 3 |
| NE | 0.887 | 0.887 | 5 |

**Table 8:** Formal data exploratory factor analysis table

| **variable** | | **item number** | factor loading **（rotated factor loading）** | | **Commonality** | **Cumulative variance interpretability** | **KMO** | **Bartlettapproximate chi square** | **p** |
| --- | --- | --- | --- | --- | --- | --- | --- | --- | --- |
| PA | problem recognition | PA1 | 0.748 |  | 0.655 | 66.607% | 0.910 | 2918.965 | 0.000 |
|  |  | PA2 | 0.836 |  | 0.724 |  |  |  |  |
|  |  | PA3 | 0.692 |  | 0.586 |  |  |  |  |
|  | Problem Involvement | PA4 |  | 0.595 | 0.602 |  |  |  |  |
|  |  | **PA5** | **0.733** |  | 0.637 |  |  |  |  |
|  |  | **PA6** |  | **0.438** | 0.571 |  |  |  |  |
|  |  | PA7 |  | 0.918 | 0.888 |  |  |  |  |
| IN | / | IN1 | 0.626 | | 0.401 | 66.308% | 0.894 | 3565.149 | 0.000 |
|  |  | IN2 | 0.841 | | 0.707 |  |  |  |  |
|  |  | IN3 | 0.869 | | 0.755 |  |  |  |  |
|  |  | IN4 | 0.849 | | 0.720 |  |  |  |  |
|  |  | IN5 | 0.876 | | 0.767 |  |  |  |  |
|  |  | IN6 | 0.799 | | 0.638 |  |  |  |  |
| GI | Group satisfaction | GI1 | 0.804 |  | 0.718 | 67.028% | 0.934 | 4664.744 | 0.000 |
|  |  | GI2 | 0.822 |  | 0.720 |  |  |  |  |
|  |  | GI3 | 0.738 |  | 0.683 |  |  |  |  |
|  |  | GI4 | 0.747 |  | 0.670 |  |  |  |  |
|  | group solidarity | GI5 |  | 0.783 | 0.643 |  |  |  |  |
|  |  | **GI6** | **0.610** | **0.471** | 0.594 |  |  |  |  |
|  |  | GI7 |  | 0.691 | 0.621 |  |  |  |  |
|  |  | GI8 |  | 0.636 | 0.659 |  |  |  |  |
|  |  | GI9 |  | 0.795 | 0.725 |  |  |  |  |
| IG | / | IG1 | 0.847 | | 0.718 | 77.995% | 0.844 | 2611.370 | 0.000 |
|  |  | IG2 | 0.886 | | 0.786 |  |  |  |  |
|  |  | IG3 | 0.910 | | 0.828 |  |  |  |  |
|  |  | IG4 | 0.888 | | 0.788 |  |  |  |  |
| SF | Sense of system justice | SF1 | 0.767 |  | 0.738 | 72.733% | 0.970 | 8711.501 | 0.000 |
|  |  | SF2 | 0.722 |  | 0.714 |  |  |  |  |
|  |  | SF3 | 0.521 |  | 0.708 |  |  |  |  |
|  |  | **SF4** | **0.493** |  | 0.668 |  |  |  |  |
|  |  | SF5 | 0.686 |  | 0.721 |  |  |  |  |
|  |  | SF6 | 0.611 |  | 0.700 |  |  |  |  |
|  |  | SF7 | 0.726 |  | 0.729 |  |  |  |  |
|  | Self justice | SF8 |  | 0.835 | 0.802 |  |  |  |  |
|  |  | SF9 |  | 0.760 | 0.757 |  |  |  |  |
|  |  | SF10 |  | 0.815 | 0.766 |  |  |  |  |
|  |  | SF11 |  | 0.672 | 0.698 |  |  |  |  |
| GE | / | GE1 | 0.848 | | 0.720 | 72.681% | 0.716 | 1006.708 | 0.000 |
|  |  | GE2 | 0.851 | | 0.725 |  |  |  |  |
|  |  | GE3 | 0.858 | | 0.736 |  |  |  |  |
| NE | / | NE1 | 0.830 | | 0.689 | 68.842% | 0.886 | 2561.334 | 0.000 |
|  |  | NE2 | 0.846 | | 0.715 |  |  |  |  |
|  |  | NE3 | 0.842 | | 0.709 |  |  |  |  |
|  |  | NE4 | 0.797 | | 0.635 |  |  |  |  |
|  |  | NE5 | 0.833 | | 0.693 |  |  |  |  |
| *p＜0.05，**p＜0.01，***p＜0.001 | | | | | | | | | |

**Table 9:** T-test table for independent samples of each variable on gender

| **variables** | **M（n=475）** | **F（n=529）** | **P** | **T** | **95% confidence interval for difference** | |
| --- | --- | --- | --- | --- | --- | --- |
|  |  |  |  |  | **lower** | **upper** |
| SF | 5.311±1.485 | 5.158±1.421 | 0.096 | 1.664 | -0.027 | 0.333 |
| PA | 3.809±0.997 | 3.84±0.892 | 0.608 | -0.513 | -0.149 | 0.087 |
| NE | 3.748±1.146 | 3.784±1.004 | 0.607 | -0.514 | -0.169 | 0.099 |
| IN | 5.134±1.307 | 4.832±1.363 | 0.000 | 3.572 | 0.136 | 0.468 |
| IG | 5.056±1.603 | 5.198±1.409 | 0.136 | -1.491 | -0.331 | 0.045 |
| GI | 3.712±0.972 | 3.837±0.865 | 0.031 | -2.157 | -0.240 | -0.011 |
| GE | 3.714±1.089 | 3.721±1.029 | 0.911 | -0.112 | -0.139 | 0.124 |

**Table 10:** Independent sample T test of each variable in registered residence registration category

| **variable** | **Urban** | **Rural** | **T** | **P** | **95% confidence interval for difference** | |
| --- | --- | --- | --- | --- | --- | --- |
|  |  |  |  |  | **lower** | **upper** |
| SF | 5.16±1.531 | 5.321±1.341 | -1.772 | 0.077 | -0.339 | 0.017 |
| PA | 3.825±0.925 | 3.827±0.967 | -0.033 | 0.973 | -0.12 | 0.116 |
| NE | 3.717±1.109 | 3.831±1.023 | -1.687 | 0.092 | -0.247 | 0.019 |
| IN | 5.02±1.376 | 4.915±1.302 | 1.228 | 0.22 | -0.063 | 0.273 |
| IG | 5.139±1.456 | 5.12±1.568 | 0.195 | 0.846 | -0.169 | 0.207 |
| GI | 3.785±0.918 | 3.768±0.921 | 0.288 | 0.773 | -0.098 | 0.132 |
| GE | 3.722±1.061 | 3.712±1.054 | 0.144 | 0.886 | -0.123 | 0.142 |

**Table 11:** Single factor ANOVA test table for cultural level of each variable

| **variable** | | **PA** | **IN** | **GI** | **IG** | **SF** | **GE** | **NE** |
| --- | --- | --- | --- | --- | --- | --- | --- | --- |
| Primary and below | mean | 4.167 | 5.767 | 3.900 | 6.300 | 5.967 | 4.100 | 4.167 |
|  | standard deviation | 0.794 | 1.067 | 1.137 | 0.775 | 1.172 | 0.784 | 0.976 |
| junior | mean | 3.843 | 5.199 | 3.881 | 5.364 | 5.353 | 3.846 | 4.021 |
|  | standard deviation | 0.928 | 1.406 | 0.929 | 1.494 | 1.270 | 0.988 | 0.945 |
| senior | mean | 3.486 | 4.823 | 3.455 | 4.882 | 5.188 | 3.638 | 3.624 |
|  | standard deviation | 1.112 | 1.395 | 0.993 | 1.565 | 1.399 | 1.228 | 1.157 |
| polytechnic | mean | 3.762 | 5.230 | 3.801 | 5.285 | 5.313 | 3.938 | 3.660 |
|  | standard deviation | 1.103 | 1.372 | 0.981 | 1.673 | 1.445 | 1.099 | 1.190 |
| undergraduate | mean | 3.958 | 4.987 | 3.871 | 5.228 | 5.444 | 3.724 | 3.858 |
|  | standard deviation | 0.849 | 1.272 | 0.865 | 1.445 | 1.412 | 1.047 | 1.061 |
| Master or above | mean | 3.896 | 4.652 | 3.798 | 4.759 | 4.568 | 3.476 | 3.548 |
|  | standard deviation | 0.732 | 1.312 | 0.805 | 1.369 | 1.578 | 0.867 | 0.956 |
| F | | 7.005 | 5.269 | 5.735 | 6.263 | 10.102 | 3.936 | 4.934 |
| P | | 0.000 | 0.000 | 0.000 | 0.000 | 0.000 | 0.002 | 0.000 |
| LSD Comparison | | 1>5,6,2,4,3 | 1>4,2,5,3,6 | 1>2,5,4,6,3 | 1>2,4,5,3,6 | 1>5,2,4,3,6 | 1>4,2,5,3,6 | 1>2,5,4,3,6 |

**Table 12:** One-way ANOVA test table of each variable on age

| **variable/age** | | **PA** | **IN** | **GI** | **IG** | **SF** | **GE** | **NE** |
| --- | --- | --- | --- | --- | --- | --- | --- | --- |
| Under 19 | mean | 4.146 | 4.878 | 3.854 | 5.317 | 5.427 | 3.683 | 3.841 |
|  | standard deviation | 0.831 | 1.359 | 0.853 | 1.532 | 1.477 | 1.161 | 1.175 |
| 20-29 | mean | 3.914 | 4.870 | 3.841 | 5.098 | 5.173 | 3.646 | 3.801 |
|  | standard deviation | 0.846 | 1.288 | 0.836 | 1.394 | 1.462 | 1.023 | 1.013 |
| 30-39 | mean | 3.754 | 5.241 | 3.708 | 5.022 | 5.389 | 3.749 | 3.670 |
|  | standard deviation | 0.981 | 1.242 | 0.945 | 1.747 | 1.423 | 1.097 | 1.094 |
| 40-49 | mean | 3.542 | 4.816 | 3.447 | 4.947 | 4.716 | 3.589 | 3.289 |
|  | standard deviation | 1.112 | 1.647 | 1.140 | 1.778 | 1.606 | 1.255 | 1.385 |
| 50-59 | mean | 3.812 | 5.139 | 3.904 | 5.410 | 5.481 | 3.975 | 4.065 |
|  | standard deviation | 0.973 | 1.346 | 0.896 | 1.333 | 1.217 | 0.934 | 0.867 |
| 60 years and over | mean | 3.240 | 4.780 | 3.360 | 5.180 | 5.180 | 3.780 | 3.560 |
|  | standard deviation | 1.268 | 1.601 | 1.221 | 1.464 | 1.761 | 1.021 | 1.139 |
| F | | 5.832 | 2.972 | 4.933 | 1.782 | 4.167 | 2.737 | 7.092 |
| P | | 0.000 | 0.011 | 0.000 | 0.114 | 0.001 | 0.018 | 0.000 |
| LSD Comparison | | 1>2,5,3,4,6 | 3>5,1,2,4,6 | 5>1,2,3,4,6 | - | 5>1,3,6,2,4 | 5>3,1,2,4,6 | 5>1,2,3,6,4 |

**Table 13:** One-way ANOVA test table of each variable on the objective level

| **objective income/variable** | | **PA** | **IN** | **GI** | **IG** | **SF** | **GE** | **NE** |
| --- | --- | --- | --- | --- | --- | --- | --- | --- |
| Very Low-Income Tier | mean | 3.955 | 4.478 | 3.665 | 4.866 | 4.920 | 3.451 | 3.545 |
|  | standard deviation | 0.914 | 1.466 | 0.886 | 1.507 | 1.596 | 1.050 | 1.154 |
| Low-income tier | mean | 3.747 | 4.972 | 3.762 | 5.071 | 5.198 | 3.736 | 3.743 |
|  | standard deviation | 0.956 | 1.284 | 0.912 | 1.503 | 1.416 | 1.057 | 1.068 |
| Middle-income group | mean | 3.867 | 5.100 | 3.811 | 5.224 | 5.322 | 3.740 | 3.870 |
|  | standard deviation | 0.929 | 1.325 | 0.904 | 1.512 | 1.438 | 1.041 | 1.000 |
| High-income tier | mean | 4.093 | 5.296 | 4.056 | 5.963 | 5.778 | 4.167 | 3.648 |
|  | standard deviation | 0.931 | 1.700 | 1.296 | 1.037 | 1.489 | 1.135 | 1.610 |
| F | | 2.803 | 6.842 | 1.608 | 4.699 | 3.604 | 4.146 | 2.989 |
| P | | 0.039 | 0.000 | 0.186 | 0.003 | 0.013 | 0.006 | 0.030 |
| LSD Comparison | | 4>1,3,2 | 4>3,2,1 | - | 4>3,2,1 | 4>3,2,1 | 4>3,2,1 | 3>2,4,1 |

**Table 14:** Single-factor ANOVA test table of each variable on subjective class identity

| **Subjective social class/variable** | | **PA** | **IN** | **GI** | **IG** | **SF** | **GE** | **NE** |
| --- | --- | --- | --- | --- | --- | --- | --- | --- |
| Upper | mean | 3.971 | 5.291 | 3.954 | 5.605 | 5.587 | 3.936 | 3.948 |
|  | standard deviation | 0.905 | 1.314 | 0.963 | 1.459 | 1.265 | 1.068 | 1.000 |
| Upper-middle | mean | 4.023 | 5.417 | 3.922 | 5.634 | 5.706 | 4.068 | 4.004 |
|  | standard deviation | 0.755 | 1.059 | 0.742 | 1.110 | 1.017 | 0.905 | 0.878 |
| Middle | mean | 3.784 | 4.888 | 3.787 | 5.072 | 5.256 | 3.724 | 3.762 |
|  | standard deviation | 0.962 | 1.350 | 0.897 | 1.517 | 1.431 | 0.985 | 1.096 |
| Lower -middle | mean | 3.705 | 4.551 | 3.614 | 4.588 | 4.585 | 3.229 | 3.484 |
|  | standard deviation | 1.027 | 1.482 | 1.054 | 1.628 | 1.741 | 1.164 | 1.173 |
| Lower | mean | 3.459 | 4.704 | 3.327 | 4.500 | 4.551 | 3.469 | 3.439 |
|  | standard deviation | 1.098 | 1.354 | 1.034 | 1.633 | 1.415 | 1.188 | 1.139 |
| F | | 6.061 | 13.897 | 6.837 | 18.343 | 21.170 | 19.442 | 8.131 |
| P | | 0.000 | 0.000 | 0.000 | 0.000 | 0.000 | 0.000 | 0.000 |
| LSD post hoc comparison | | 5>4,3,1,2 | 4>3,5,1,2 | 5>4,3,1,4 | 5>4,3,2,1 | 5>4,3,1,2 | 4>5,3,1,2 | 5>4,3,1,2 |

**Table 15:** Judgement Table for Fitting Indicators to SEMs

| **Fitting Indicator** | **Actual metrics** | **Fit or not** |
| --- | --- | --- |
| CMIN | 2110.04 | Fit |
| DF | 754 | Fit |
| P | 0.000 | Fit |
| CMIN/DF | 2.798 | Fit |
| GFI | 0.902 | Fit |
| AGFI | 0.888 | Fit |
| NFI | 0.924 | Fit |
| RFI | 0.918 | Fit |
| IFI | 0.950 | Fit |
| TLI | 0.946 | Fit |
| CFI | 0.950 | Fit |
| PNFI | 0.850 | Fit |
| PCFI | 0.874 | Fit |
| RMSEA | 0.042 | Fit |

**Table 16:** Percentage of Intermediation Effects

| **Pathway** | | | **Efficiency ratio** |
| --- | --- | --- | --- |
| A2 | PA→IN→SF→NE | X1*X9*X11 | 9.43% |
| A5 | PA→GI→SF→NE | X3*X8*X11 | 9.74% |
| A6 | PA→GI→GE→NE | X3*X7*X13 | 15.16% |
| A8 | IG→IN→SF→NE | X5*X9*X11 | 7.41% |
| A10 | IG→GI→SF→NE | X6*X8*X11 | 9.05% |
| A11 | IG→GI→GE→NE | X6*X7*X13 | 13.77% |
| A13 | IN→SF→NE | X9*X11 | 11.50% |
| A15 | GI→SF→NE | X8*X11 | 11.09% |
| A16 | PA→GI→GE | X3*X7 | 60.52% |
| A17 | GI→GE→NE | X7*X13 | 22.72% |

**Table 17:** Factor Load and cross-matching Table of Question Items

| **items** | **factor** | **items** | **factor** |
| --- | --- | --- | --- |
| IG3 | 0.889 | GI4 | 0.762 |
| IG2 | 0.848 | GI3 | 0.78 |
| IG4 | 0.847 | GI1 | 0.768 |
| PA5 | 0.743 | GE3 | 0.783 |
| PA2 | 0.736 | GE2 | 0.765 |
| PA6 | 0.722 | GE1 | 0.757 |
| **cross-matching** | | | |
| int1 | IG3-PA6 | int4 | GE3-GI4 |
| int2 | IG2-PA3 | int5 | GE2-GI2 |
| int3 | IG4-PA7 | int6 | GE1-GI5 |

**Table 18:** Latent variable moderating effects

| **Moderating Effect 1: Moderating Role of IG in PA and IN** | | | | | | | |
| --- | --- | --- | --- | --- | --- | --- | --- |
| **pathway** | | | **Estimate** | **S.E.** | **C.R.** | **P** | **Label** |
| IG | ---> | IN | 0.176 | 0.035 | 5.071 | *** | m |
| PA | ---> | IN | 0.932 | 0.084 | 11.137 | *** | x |
| intPAIG | ---> | IN | 0.047 | 0.036 | 1.310 | 0.190 | xm |
| **Latent variable moderating effect 2: Moderating role of IG in PA and GI** | | | | | | | |
| **pathway** | | | **Estimate** | **S.E.** | **C.R.** | **P** | **Label** |
| PA | ---> | GI | 0.488 | 0.043 | 11.306 | *** | x |
| IG | ---> | GI | 0.206 | 0.020 | 10.456 | *** | m |
| intPAIG | ---> | GI | 0.027 | 0.019 | 1.411 | 0.158 | xm |

| **Latent variable moderating effect 3: Moderating role of IG in PA and SF** | | | | | | | |
| --- | --- | --- | --- | --- | --- | --- | --- |
| **pathway** | | | **Estimate** | **S.E.** | **C.R.** | **P** | **Label** |
| PA | ---> | SF | 0.384 | 0.069 | 5.611 | *** | x |
| IG | ---> | SF | 0.404 | 0.034 | 11.742 | *** | m |
| intPAIG | ---> | SF | 0.048 | 0.034 | 1.404 | 0.160 | xm |
| **Latent variable moderating effect 4: Moderating role of IG in PA and GE** | | | | | | | |
| **pathway** | | | **Estimate** | **S.E.** | **C.R.** | **P** | **Label** |
| PA | ---> | GE | 0.401 | 0.049 | 8.225 | *** | x |
| IG | ---> | GE | 0.248 | 0.023 | 10.669 | *** | m |
| intPAIG | ---> | GE | 0.021 | 0.023 | 0.924 | 0.356 | xm |
| **Latent variable moderating effect 5: Moderating role of GI in GE and NE** | | | | | | | |
| **pathway** | | | **Estimate** | **S.E.** | **C.R.** | **P** | **Label** |
| GE | ---> | NE | 0.466 | 0.047 | 9.935 | *** | x |
| GI | ---> | NE | 0.534 | 0.061 | 8.784 | *** | m |
| intGEGI | ---> | NE | 0.266 | 0.053 | 5.017 | *** | xm |

*p＜0.05，**p＜0.01，*** p＜0.001
