# Supplementary material for: Rational resistance amidst gentle breeze and mild rain: Examining online collective behavior among the Chinese public using the elaborated social identity model
Source: PLoS One. 2024 May 24;19(5):e0303603. doi: 10.1371/journal.pone.0303603 (PMC11125461; doi:10.1371/journal.pone.0303603)
Supplement: S2 File — (DOCX) [file pone.0303603.s003.docx]

**Table 13:** One-way ANOVA test table of each variable on the objective level

| **objective income/variable** | | **PA** | **IN** | **GI** | **IG** | **SF** | **GE** | **NE** |
| --- | --- | --- | --- | --- | --- | --- | --- | --- |
| Very Low-Income Tier | mean | 3.955 | 4.478 | 3.665 | 4.866 | 4.920 | 3.451 | 3.545 |
|  | standard deviation | 0.914 | 1.466 | 0.886 | 1.507 | 1.596 | 1.050 | 1.154 |
| Low-income tier | mean | 3.747 | 4.972 | 3.762 | 5.071 | 5.198 | 3.736 | 3.743 |
|  | standard deviation | 0.956 | 1.284 | 0.912 | 1.503 | 1.416 | 1.057 | 1.068 |
| Middle-income group | mean | 3.867 | 5.100 | 3.811 | 5.224 | 5.322 | 3.740 | 3.870 |
|  | standard deviation | 0.929 | 1.325 | 0.904 | 1.512 | 1.438 | 1.041 | 1.000 |
| High-income tier | mean | 4.093 | 5.296 | 4.056 | 5.963 | 5.778 | 4.167 | 3.648 |
|  | standard deviation | 0.931 | 1.700 | 1.296 | 1.037 | 1.489 | 1.135 | 1.610 |
| F | | 2.803 | 6.842 | 1.608 | 4.699 | 3.604 | 4.146 | 2.989 |
| P | | 0.039 | 0.000 | 0.186 | 0.003 | 0.013 | 0.006 | 0.030 |
| LSD Comparison | | 4>1,3,2 | 4>3,2,1 | - | 4>3,2,1 | 4>3,2,1 | 4>3,2,1 | 3>2,4,1 |

**Table 14:** Single-factor ANOVA test table of each variable on subjective class identity

| **Subjective social class/variable** | | **PA** | **IN** | **GI** | **IG** | **SF** | **GE** | **NE** |
| --- | --- | --- | --- | --- | --- | --- | --- | --- |
| Upper | mean | 3.971 | 5.291 | 3.954 | 5.605 | 5.587 | 3.936 | 3.948 |
|  | standard deviation | 0.905 | 1.314 | 0.963 | 1.459 | 1.265 | 1.068 | 1.000 |
| Upper-middle | mean | 4.023 | 5.417 | 3.922 | 5.634 | 5.706 | 4.068 | 4.004 |
|  | standard deviation | 0.755 | 1.059 | 0.742 | 1.110 | 1.017 | 0.905 | 0.878 |
| Middle | mean | 3.784 | 4.888 | 3.787 | 5.072 | 5.256 | 3.724 | 3.762 |
|  | standard deviation | 0.962 | 1.350 | 0.897 | 1.517 | 1.431 | 0.985 | 1.096 |
| Lower -middle | mean | 3.705 | 4.551 | 3.614 | 4.588 | 4.585 | 3.229 | 3.484 |
|  | standard deviation | 1.027 | 1.482 | 1.054 | 1.628 | 1.741 | 1.164 | 1.173 |
| Lower | mean | 3.459 | 4.704 | 3.327 | 4.500 | 4.551 | 3.469 | 3.439 |
|  | standard deviation | 1.098 | 1.354 | 1.034 | 1.633 | 1.415 | 1.188 | 1.139 |
| F | | 6.061 | 13.897 | 6.837 | 18.343 | 21.170 | 19.442 | 8.131 |
| P | | 0.000 | 0.000 | 0.000 | 0.000 | 0.000 | 0.000 | 0.000 |
| LSD post hoc comparison | | 5>4,3,1,2 | 4>3,5,1,2 | 5>4,3,1,4 | 5>4,3,2,1 | 5>4,3,1,2 | 4>5,3,1,2 | 5>4,3,1,2 |

**Table 15:** Judgement Table for Fitting Indicators to SEMs

| **Fitting Indicator** | **Actual metrics** | **Fit or not** |
| --- | --- | --- |
| CMIN | 2110.04 | Fit |
| DF | 754 | Fit |
| P | 0.000 | Fit |
| CMIN/DF | 2.798 | Fit |
| GFI | 0.902 | Fit |
| AGFI | 0.888 | Fit |
| NFI | 0.924 | Fit |
| RFI | 0.918 | Fit |
| IFI | 0.950 | Fit |
| TLI | 0.946 | Fit |
| CFI | 0.950 | Fit |
| PNFI | 0.850 | Fit |
| PCFI | 0.874 | Fit |
| RMSEA | 0.042 | Fit |

**Table 16:** Percentage of Intermediation Effects

| **Pathway** | | | **Efficiency ratio** |
| --- | --- | --- | --- |
| A2 | PA→IN→SF→NE | X1*X9*X11 | 9.43% |
| A5 | PA→GI→SF→NE | X3*X8*X11 | 9.74% |
| A6 | PA→GI→GE→NE | X3*X7*X13 | 15.16% |
| A8 | IG→IN→SF→NE | X5*X9*X11 | 7.41% |
| A10 | IG→GI→SF→NE | X6*X8*X11 | 9.05% |
| A11 | IG→GI→GE→NE | X6*X7*X13 | 13.77% |
| A13 | IN→SF→NE | X9*X11 | 11.50% |
| A15 | GI→SF→NE | X8*X11 | 11.09% |
| A16 | PA→GI→GE | X3*X7 | 60.52% |
| A17 | GI→GE→NE | X7*X13 | 22.72% |

**Table 17:** Factor Load and cross-matching Table of Question Items

| **items** | **factor** | **items** | **factor** |
| --- | --- | --- | --- |
| IG3 | 0.889 | GI4 | 0.762 |
| IG2 | 0.848 | GI3 | 0.78 |
| IG4 | 0.847 | GI1 | 0.768 |
| PA5 | 0.743 | GE3 | 0.783 |
| PA2 | 0.736 | GE2 | 0.765 |
| PA6 | 0.722 | GE1 | 0.757 |
| **cross-matching** | | | |
| int1 | IG3-PA6 | int4 | GE3-GI4 |
| int2 | IG2-PA3 | int5 | GE2-GI2 |
| int3 | IG4-PA7 | int6 | GE1-GI5 |

**Table 18:** Latent variable moderating effects

| **Moderating Effect 1: Moderating Role of IG in PA and IN** | | | | | | | |
| --- | --- | --- | --- | --- | --- | --- | --- |
| **pathway** | | | **Estimate** | **S.E.** | **C.R.** | **P** | **Label** |
| IG | ---> | IN | 0.176 | 0.035 | 5.071 | *** | m |
| PA | ---> | IN | 0.932 | 0.084 | 11.137 | *** | x |
| intPAIG | ---> | IN | 0.047 | 0.036 | 1.310 | 0.190 | xm |
| **Latent variable moderating effect 2: Moderating role of IG in PA and GI** | | | | | | | |
| **pathway** | | | **Estimate** | **S.E.** | **C.R.** | **P** | **Label** |
| PA | ---> | GI | 0.488 | 0.043 | 11.306 | *** | x |
| IG | ---> | GI | 0.206 | 0.020 | 10.456 | *** | m |
| intPAIG | ---> | GI | 0.027 | 0.019 | 1.411 | 0.158 | xm |

| **Latent variable moderating effect 3: Moderating role of IG in PA and SF** | | | | | | | |
| --- | --- | --- | --- | --- | --- | --- | --- |
| **pathway** | | | **Estimate** | **S.E.** | **C.R.** | **P** | **Label** |
| PA | ---> | SF | 0.384 | 0.069 | 5.611 | *** | x |
| IG | ---> | SF | 0.404 | 0.034 | 11.742 | *** | m |
| intPAIG | ---> | SF | 0.048 | 0.034 | 1.404 | 0.160 | xm |
| **Latent variable moderating effect 4: Moderating role of IG in PA and GE** | | | | | | | |
| **pathway** | | | **Estimate** | **S.E.** | **C.R.** | **P** | **Label** |
| PA | ---> | GE | 0.401 | 0.049 | 8.225 | *** | x |
| IG | ---> | GE | 0.248 | 0.023 | 10.669 | *** | m |
| intPAIG | ---> | GE | 0.021 | 0.023 | 0.924 | 0.356 | xm |
| **Latent variable moderating effect 5: Moderating role of GI in GE and NE** | | | | | | | |
| **pathway** | | | **Estimate** | **S.E.** | **C.R.** | **P** | **Label** |
| GE | ---> | NE | 0.466 | 0.047 | 9.935 | *** | x |
| GI | ---> | NE | 0.534 | 0.061 | 8.784 | *** | m |
| intGEGI | ---> | NE | 0.266 | 0.053 | 5.017 | *** | xm |

*p＜0.05，**p＜0.01，*** p＜0.001
